# N:P stoichiometric changes via species turnover in arid versus saline desert environments

**DOI:** 10.1002/ece3.6395

**Published:** 2020-05-30

**Authors:** Yan‐Ming Gong, Hong‐Bo Ling, Yue Chen, Jing Cao, Zhen‐Jie Guo, Guang‐Hui Lv

**Affiliations:** ^1^ State Key Laboratory of Desert and Oasis Ecology Xinjiang Institute of Ecology and Geography Chinese Academy of Sciences (CAS) Urumqi China; ^2^ Xinjiang Key Laboratory of Oasis Ecology Xinjiang University Urumqi China

**Keywords:** community stoichiometry, extreme environment, intraspecies variation, nutrient concentration, species turnover

## Abstract

1. Aridity and salinity have a key role in driving physiological and ecological processes in desert ecosystems. However, how community‐scale foliar nutrients respond to aridity and salinity, and how these responses might vary with community composition along aridity and salinity gradients is unclear. We hypothesize that the response will be a shift in community stoichiometric values resulting from nutrient variability of shared species and unique species (site‐specific species), but little research has addressed the relative contribution of either component.
2. We analyzed the community‐scale stoichiometric response of a desert community of perennial plants along an aridity and salinity transect by focusing on foliar nitrogen (N) and phosphorous (P) concentrations and N:P ratios. After evaluating the shared and unique species variability, we determined their relative contribution to the community stoichiometric response to aridity and salinity, reflected by changes in nonweighted and weighted community‐average values.
3. Community‐scale stoichiometry decreased significantly under aridity and salinity, with significantly consistent changes in nonweighted and weighted community‐average stoichiometry for most shared and unique species measurements. The relative contribution of unique species shifts to the changes in community stoichiometry was greater (15%–77%) than the relative contribution of shared species shifts (7%–45%), excluding the change in weighted P concentration under aridity. Thus, the shifts of unique species amplified the community stoichiometric response to environmental changes.
4. *Synthesis*. These results highlighted the need for a more in‐depth consideration of shared and unique species variability to understand and predict the effects of environmental change on the stoichiometry of plant communities. Although variation in community stoichiometry can be expected under extreme aridity and salinity conditions, changes of unique species could be a more important driver of the stoichiometric response of plant communities.

## Graphical abstract

These results highlighted the need for a more in‐depth consideration of intra‐ and interspecies variability to understand and predict the effects of environmental change on the stoichiometry of plant communities. Although variation in community stoichiometry can be expected under extreme aridity and salinity conditions, changes of unique species could be a more important driver of the stoichiometric response of plant communities.

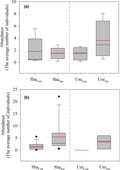

## 1 INTRODUCTION

Nitrogen (N) and phosphorus (P) are two vital elements for plant vegetative growth and reproduction and plant–soil trophic feedbacks (Cornelissen et al., 2003; Elser, Fagan, Kerkhoff, Swenson, & Enquist, 2010; Sardans et al., 2017). In particular, leaf N and P concentrations and their stoichiometric relationship can determine the photosynthesis, nutrient‐use strategies, competitive ability, energy metabolism, and net primary production of plants (Elser et al., 2000; Minden & Venterink, 2019; Sardans et al., 2015; Tian et al., 2019). Based on stoichiometric homeostasis, the stability of plant nutrient concentrations as species‐specific functional traits is under genetic control, despite fluctuations in nutrient supplies from the habitat (Demars & Edwards, 2007; Elser, 2002; Elser et al., 2010). The relative contributions of intra‐ and interspecific N and P variations to shifts in community stoichiometry under environmental change have implications for the resistance of plant communities to such change (Albert et al., 2010; Reich & Oleksyn, 2004; Violle et al., 2012). The higher relative importance of intraspecific variation implies a higher adaptation ability of plant species and contributes to higher resistance. In contrast, the higher relative importance of interspecific variation indicates greater species turnover and, thus, implies the lower resistance of plant communities to environmental change (Kichenin, Wardle, Peltzer, Morse, & Freschet, 2013; Lv et al., 2018). Therefore, understanding plant inter‐ and intraspecific variation in N:P stoichiometry is a fundamental challenge in plant ecophysiology (Osnas et al., 2018; Zuo et al., 2016).

To date, research has focused on studying the adaptability and plasticity of N:P stoichiometry in regard to species turnover at the community level (Luo et al., 2018; Raffard, Santoul, Cucherousset, & Blanchet, 2019). The changes in community N:P stoichiometry can be caused by the variations among plant individuals in nutrient concentrations, species abundance, and richness change, among others (Albert et al., 2010; Leps, de Bello, Smilauer, & Dolezal, 2011; Violle et al., 2012). Therefore, quantifying the responses of N and P to environmental change could help us to understand the relative contribution of species turnover to such change. For example, a recent study showed that increased nutrient concentrations in the plant canopy in response to aridity is a more major contributor to the effects of species turnover compared with intraspecific variation (Luo et al., 2018). For community‐level functional traits, Zuo et al. (2016) concluded that there was a large relative contribution of interspecific variation to the community response to environmental change compared with intraspecific variation. The relative importance of interspecific trait variation can increase along a wide environmental gradient (Albert et al., 2010; Kichenin et al., 2013; Vila‐Cabrera, Martinez‐Vilalta, & Retana, 2015). Thus, we suggest that, driven by the environment, interspecific variation in plant community stoichiometry is mainly due to changes in species richness, species abundance, or both (Guiz et al., 2018; Jung et al., 2014). For this reason, we considered to divide the plant species in communities into two categories, namely shared species and unique species. On this basis, the community's stoichiometric change trend under the influence of drought and salinity is analyzed by weighting relative abundance.

There has been growing interest in the relative importance of species turnover variation versus intraspecific variability in vascular plants in determining total community‐level stoichiometric variation across communities and environmental gradients (Asplund & Wardle, 2014; Luo et al., 2018). In parallel, ecologists have proposed and utilized several practical methods to evaluate the relative contributions of both components (Hou et al., 2019; Jackson, Peltzer, & Wardle, 2013; Jung et al., 2014; Kichenin et al., 2013; Leps et al., 2011; Luo et al., 2018; Zuo et al., 2016). These methods emphasize the effects of variation in individual plant nutrient concentration and magnitude of relative abundance (or relative biomass) on community stoichiometry under environmental change. However, the influence of species emergence and disappearance caused by environmental filtering on community‐level stoichiometry has not yet been clearly identified. Thus, here we present additional insights for understanding the effects of unique (site‐specific) and shared species shifts in community stoichiometry in response to environmental change. Furthermore, we define unique and shared species stoichiometric differences between communities as stoichiometric variation of species turnover under different habitat conditions, respectively. Community‐level mean stoichiometric value variations are often counted by nonweighted (CM) and relative abundance weighted (CWM) calculations (Lv et al., 2018; Volf et al., 2016; Zuo et al., 2016). For CM, a higher relative contribution of intraspecific trait variability indicates a greater resistance of plant community composition to such change (Mason et al., 2012), whereas, for CWM, it implies the greater resistance of both community composition and the relative abundance of dominant species (Violle et al., 2007). Thus, comparing the responses of CM and CWM to the same treatments would help clarify the role of subdominant and dominant species to the stoichiometric responses to environmental change.

Under the influence of climate change and anthropogenic disturbance, the effects of aridity in terrestrial ecosystems are likely to intensify (Feng & Fu, 2013; Gao & Giorgi, 2008). In addition, in response to agricultural measures, such as irrigation, terrestrial saline‐alkali areas are also rapidly increasing worldwide (Ghassemi, Close, & Kellett, 1997). In arid areas, aridity and salinity affect both plant growth and community structure by changing the soil physical and chemical properties (He & Dijkstra, 2014; Sistla & Schimel, 2012). Aridity inhibits the mineralization of soil nutrients and decomposition of organic matter, thus affecting the effective utilization of N and P by plants (Bertiller, Sain, Carrera, & Vargas, 2005; Guiz et al., 2016; Hall, Smith, Lytle, & Leibold, 2005; Hobbie & Gough, 2002). High soil salt content can also negatively affect plant nutrient uptake (Gong, Lv, Guo, Chen, & Cao, 2017). However, N and P are involved in many physiological and ecological processes in plants, such as photosynthesis, signal transduction, and energy storage (Elser et al., 2007). Hence, a clear understanding of the effects of increasing aridity and salinity on the biogeochemistry of N and P is needed (Sardans & Penuelas, 2012). For instance, the community nutrient concentration might respond to short‐term aridity by changing relative biomass (or abundance) and intraspecific variation, while responding to longer‐term aridity through species turnover (Lajoie & Vellend, 2015; Luo et al., 2018; Sandel et al., 2010; Volf et al., 2016).

In this study, we focus on plant N and P concentrations and N:P ratios, all of which are important for biomass production and environmental suitability. We investigated the relative contributions of unique and shared species to the N:P stoichiometry responses of a desert plant community to aridity and salinity stress. Previous research showed that aridity and salinity reduced the leaf N and P concentrations across species and community scales in local dryland vegetation (Gong et al., 2017). Furthermore, we hypothesize that shifts in community N:P stoichiometry under aridity and salinity filtering are mainly driven by the relative contribution of unique (site‐specific) species rather than by more shared species. Thus, ignoring the presence of unique species in the community would underestimate the variation of community stoichiometry in response to environmental changes, even though such species are often not the dominant species in plant community in terms of their abundance.

## 2 MATERIALS AND METHODS

### 2.1 Field sampling and measurements

**FIGURE 1:**
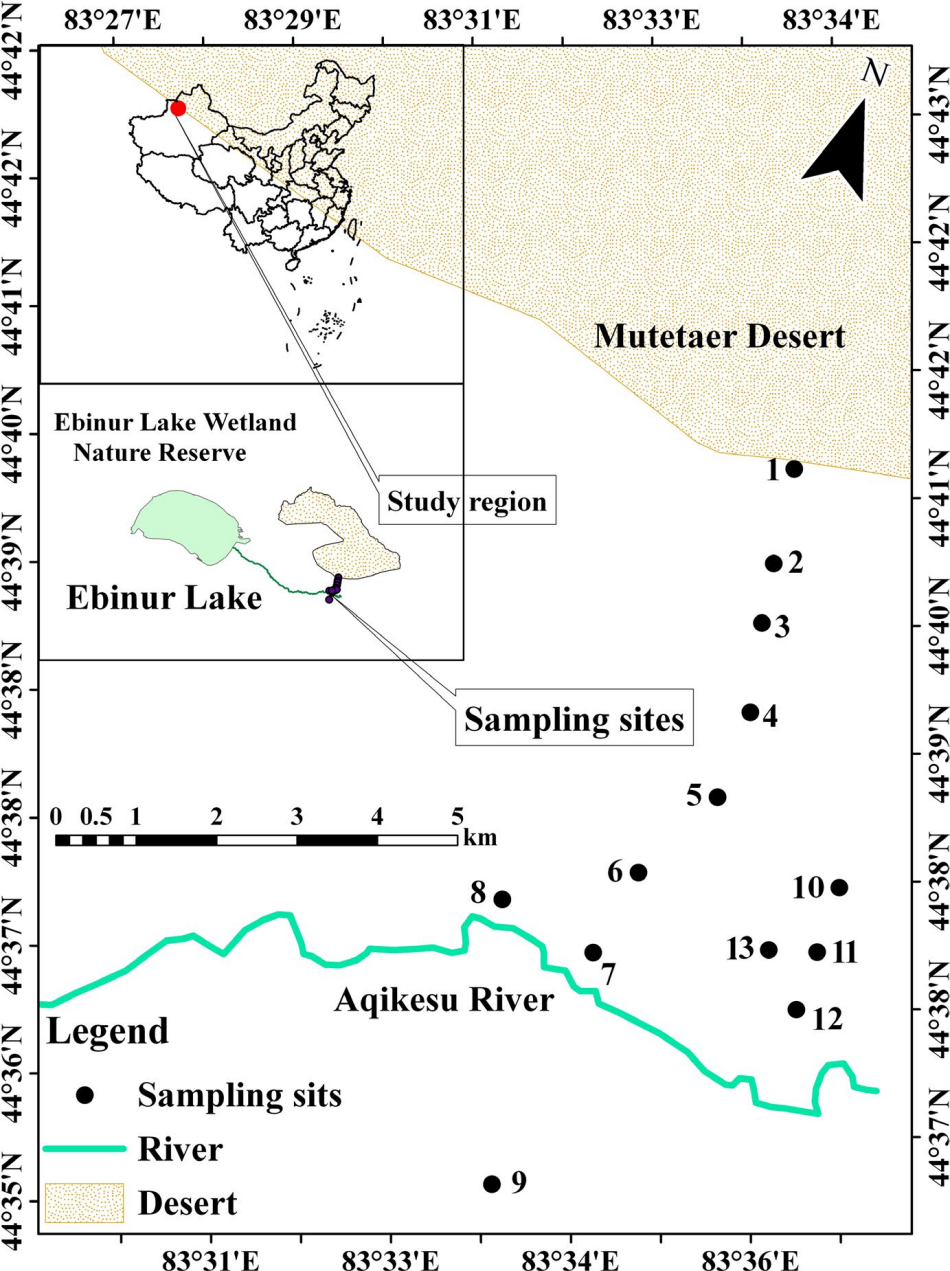
Sampling sites. An 8 km long transect was sampled in the Ebinur Lake Wetland Nature Reserve in Xinjiang Uygur Autonomous Region of China. A total of 13 sampling sites were selected along this gradient. Dry sampling sites were No. 1–3, Humid‐saline sampling sites were No. 4–9, Humid‐non saline sampling sites were No. 10–13. The vector map of China was obtained by vectorizing the Chinese map (Approval Number: GS (2016) No. 2893) supervised by the Ministry of Land and Resources of China. This figure was originally generated by the software ArcGIS 10.0 (http://www.esri.com/software/arcgis/arcgisonline)

We selected a study area with gentle topography and homogeneous continental climate to analyze the influence of environmental change on community stoichiometry, and surveyed 130 plots at 13 sites along an ~8‐km transect in the Ebinur Lake Wetland Nature Reserve, Xinjiang Uygur Autonomous Region, China (Figure 1). Soil type is predominantly desert soil belong to the Kastanozem soil group in the Food and Agriculture Organization classification system. The sampling sites selected were deemed to be representative of natural conditions without any grazing activity or exposure to other anthropogenic disturbances. Thirteen sampling sites were investigated along the transect with about 1‐km gap between each sampling site (see Figure 1). Sampling locations were GPS referenced with latitude, longitude, and elevation (eTrex Venture).

At each of the thirteen sites, ten 10 m × 10 m main plots were selected in a direction perpendicular to the transect, and each plot was separated from the next by an interval of 10 m. Collection and measurement of samples were conducted during early July, 2015 (Sampling in July, 2014 on sites 10–13). The abundance of species was recorded in each plot. Fresh and mature foliar samples were collected from five to ten individuals of each species in each site and then stored in separate paper bags (a paper bag per sample). Leaves that were similar in terms of their size, shape, and color for each species were sampled. To reduce the influences of dust or soil, foliar samples were rinsed with deionized water at least twice. Plant materials were dried at 105°C for 30 min in a portable drying oven to minimize respiration and decomposition and were later completely oven dried at 70°C to a constant weight in the laboratory. In total, 357 plant samples were collected, belonging to 18 plant species across the thirteen study sites. After removing the litter layer, soil samples were randomly collected at 0–100 cm soil depths with three replicates at each sampling site. Subsamples of each soil sample were stored at 4°C immediately after collection to determine the initial gravimetric moisture content. This was measured by drying the samples at 105°C for 48 hr to a constant weight (Gong et al., 2017).

Dried plant and soil materials were ground to pass through a 1‐mm sieve (Retsch MM 400; Retsch). Leaf N concentration was analyzed with a PE‐2400 CHN analyzer (Perkin‐Elmer). Leaf P concentration was measured colorimetrically after H_2_SO_4_‐H_2_O_2_‐HF digestion using the molybdate/stannous chloride method. The ratios of available N:P were calculated from these variables at a sample level. Soil total N was analyzed with a Kjeltec System 2300 Analyzer Unit (Tecator, Höganäs, Sweden). Soil total P content was determined with the molybdate/ascorbic acid blue method after digestion with HClO_4_ and H_2_SO_4_ acid. Soil soluble salt content was obtained using the weight method, in which the soil extraction liquid was dried, followed by removal of the organic matter from the dry residue using H_2_O_2_, and the resulting liquid dried again at 105–110°C and then weighed.

### 2.2 Calculations and statistical analyses

Before the statistical analyses, all variables in regard to each species were averaged at the plot level and all variables for the soil samples were averaged at the site level. Data were tested for normality using the Kolmogorov–Smirnov test and for equality of error variance using Levene's test. Plant foliar N and P concentrations were defined as the community‐weighted mean values (CWM) weighted by the relative abundance of each species in each plot (Kichenin et al., 2013). Community‐nonweighted mean values were calculated as the mean of all species in each plot (Mason et al., 2012).

Given that changes in foliar nutrient concentrations can be attributed to both unique (site‐specific) species and shared species under aridity and salinity stresses, their relative contributions were disentangled using Equations (1) and (2) (Jung et al., 2014):

(1) $$C_{\mathrm{AriUni}}=\left(N_{\mathrm{AriUni}}-N_{\mathrm{ConUni}}\right)/N_{\mathrm{ConUni}},C_{\mathrm{AriSha}}=\left(N_{\mathrm{AriSha}}-N_{\mathrm{ConSha}}\right)/N_{\mathrm{ConSha}}\;\text{and}\;C_{\mathrm{AriTot}}=\left(N_{\mathrm{AriTot}}-N_{\mathrm{ConSha}}\right)/N_{\mathrm{ConTot}}\;\text{for}\;\text{aridity}\;\text{filtering}$$

(2) $$C_{\mathrm{SalUni}}=\left(N_{\mathrm{SalUni}}-N_{\mathrm{ConUni}}\right)/N_{\mathrm{ConUni}},C_{\mathrm{SalSha}}=\left(N_{\mathrm{SalSha}}-N_{\mathrm{ConSha}}\right)/N_{\mathrm{ConSha}}\;\text{and}\;C_{\mathrm{SalTot}}=\left(N_{\mathrm{SalTot}}-N_{\mathrm{SalTot}}\right)/N_{\mathrm{ConTot}}\;\text{for}\;\text{salinity}\;\text{filtering}$$

*N_AriUni_*, *N_AriSha_*, *N_AriTot_* and *N_ConUni_*, *N_ConSha_*_,_ *N_ConSha_* are the mean foliar nutrient concentrations of unique species, shared species, and all the species in the aridity and control sites, respectively. *N_SalUni_*, *N_SalSha_ N_SalTot_* and *N_ConUni_*, *N_ConSha_*_,_ *N_ConTot_* are the mean foliar nutrient concentrations of unique species, shared species, and all the species in the salinity and control sites, respectively. Furthermore, a comparison between the saline and control sites showed that there was no unique species in the control sites, so the value of *N_ConUni_* was replaced by the average value of the all the species. *C_AriUni_*, *C_AriSha_*, and *C_AriTot_* represent the isolated effects of the changes in unique species, shared species, and all the species, respectively, in driving the foliar nutritional responses to aridity filtering. This description is also consistent with *C_SalUni_*, *C_SalSha_*, and *C_SalTot_* under salinity filtering.

Through principal component analysis (PCA) of the environmental data, sites 1–4, sites 5–9, and sites 10–13 were defined as arid sites (low soil salt content), saline sites (humid‐saline), and control sites (humid‐nonsaline), respectively (Figure S1). One‐way analysis of variance (ANOVA) was used to examine the differences in the weighted and nonweighted community‐average stoichiometry among the individual plants and differences in soil properties from the arid sites, saline sites, and control sites. The significance level was set at *p* < .05. All statistical analyses were conducted using the statistical package SPSS (PASW statistics 21.0; IBM Corporation, Armonk, NY, USA) and SigmaPlot 12.5 (SyStat Software Inc.). PCA was conducted using CANOCO 5.0 (Microcomputer Power).

By comparing with humid‐nonsaline sites and respectively analyzing the community species composition of arid and saline sites, eight species were unique to arid sites, including *Tamarix ramosissima* and *Karelinia caspica*, and four species were unique to saline sites, namely *Poacynum hendersonii*, *Calligonum ebinuricum*, *Seriphidium santolinum*, and *Reaumuria soongorica*. The shared species of the two sites were *Haloxylon ammodendron*, *Alhagi sparsifolia*, *Nitraria sibirica*, *Phragmites australis*, and *Populus euphratica* (Table 1). Furthermore, a comparison between the saline and control sites showed that there was no unique species in the control sites, and the species unique to the saline sites were *Poacynum hendersonii*, *Reaumuria soongorica*, and *Halostachys caspica*. All the number of shared species in the two sites was 13 (Table 2).

**TABLE 1:** Distribution of unique species and shared species between the control and arid transect sites

Site	Species (abundance)	
	Unique species (8)	Shared species (5)
Control	Tr (1.19), Kc (0.19), Kf (2.61), Hh (1.92), Av (2.61), Sm (0.89), Gu (0.14), Hs (1.92)	Ha (5.55), As (2.20), Ns (0.25), Pa (0.50), Pe (0.50)
Arid	Ph (2.75), Ce (3.01), Ss (8.08), Rs (0.54)	Ha (1.78), As (1.03), Ns (1.50), Pa (2.86), Pe (0.19)

Note

As, *Alhagi sparsifolia*. Av, *Apocynum venetum*. Ce, *Calligonum ebinuricum*. Gu, *Glycyrrhiza uralensis*. Ha, *Haloxylon ammodendron*. Hc, *Halostachys caspica*. Hh, *Halimodendron halodendron*. Hs, *Halocnemum strobilaceum*. Kc, *Karelinia caspica*. Kf, *Kalidium foliatum*. Ns, *Nitraria sibirica*. Pa, *Phragmites australis*. Pe, *Populus euphratica*. Ph, *Poacynum hendersonii*. Rs, *Reaumuria soongorica*. Sm, *Suaeda microphylla*. Ss, *Seriphidium santolinum*. Tr, *Tamarix ramosissima*. Abundance: average number of individuals per 100 square meters.

**TABLE 2:** Distribution of unique species and shared species between the control and saline transect sites

Site	Species (abundance)	
	Unique species (0)	Shared species (13)
Control		Ha (5.55), As (2.20), Ns (0.25), Pa (0.50), Pe (0.50), Tr (1.19), Kc (0.19), Kf (2.61), Hh (1.92), Av (2.61), Sm (0.89), Gu (0.14), Hs (1.92)
Saline	Ph (5.96), Rs (3.68), Hc (0.42)	Ha (0.64), As (8.84), Ns (2.82), Pa (22.10), Pe (1.66), Tr (2.06), Kc (5.24), Kf (4.98), Hh (2.32), Av (2.08), Sm (0.56), Gu (3.84), Hs (13.54)

Note

As, *Alhagi sparsifolia*. Av, *Apocynum venetum*. Ce, *Calligonum ebinuricum*. Gu, *Glycyrrhiza uralensis*. Ha, *Haloxylon ammodendron*. Hc, *Halostachys caspica*. Hh, *Halimodendron halodendron*. Hs, *Halocnemum strobilaceum*. Kc, *Karelinia caspica*. Kf, *Kalidium foliatum*. Ns, *Nitraria sibirica*. Pa, *Phragmites australis*. Pe, *Populus euphratica*. Ph, *Poacynum hendersonii*. Rs, *Reaumuria soongorica*. Sm, *Suaeda microphylla*. Ss, *Seriphidium santolinum*. Tr, *Tamarix ramosissima*. Abundance: average number of individuals per 100 square meters.

## 3 RESULTS

### 3.1 Soil properties, unique, and shared species

According to the PCA results, 13 sites were classified as either arid, saline, and humid‐nonsaline (control sites) habitats along a transect in the temperate desert (Figure S1). Soil water content in the aridity habit was significantly lower than that in saline and control habitats (*p* < .05), and soil salt content in the saline habitat was significantly higher than that in arid and control habitats (*p* < .05, Figure S2). Furthermore, soil total phosphorus (STP) and soil pH in the arid and saline habitats were significantly greater than in the control habitat (*p* < .05), respectively (Figure S3). There was no significant difference in soil organic carbon (SOC) and soil total nitrogen (STN) between the three habitats (Figure S3).

**FIGURE 2:**
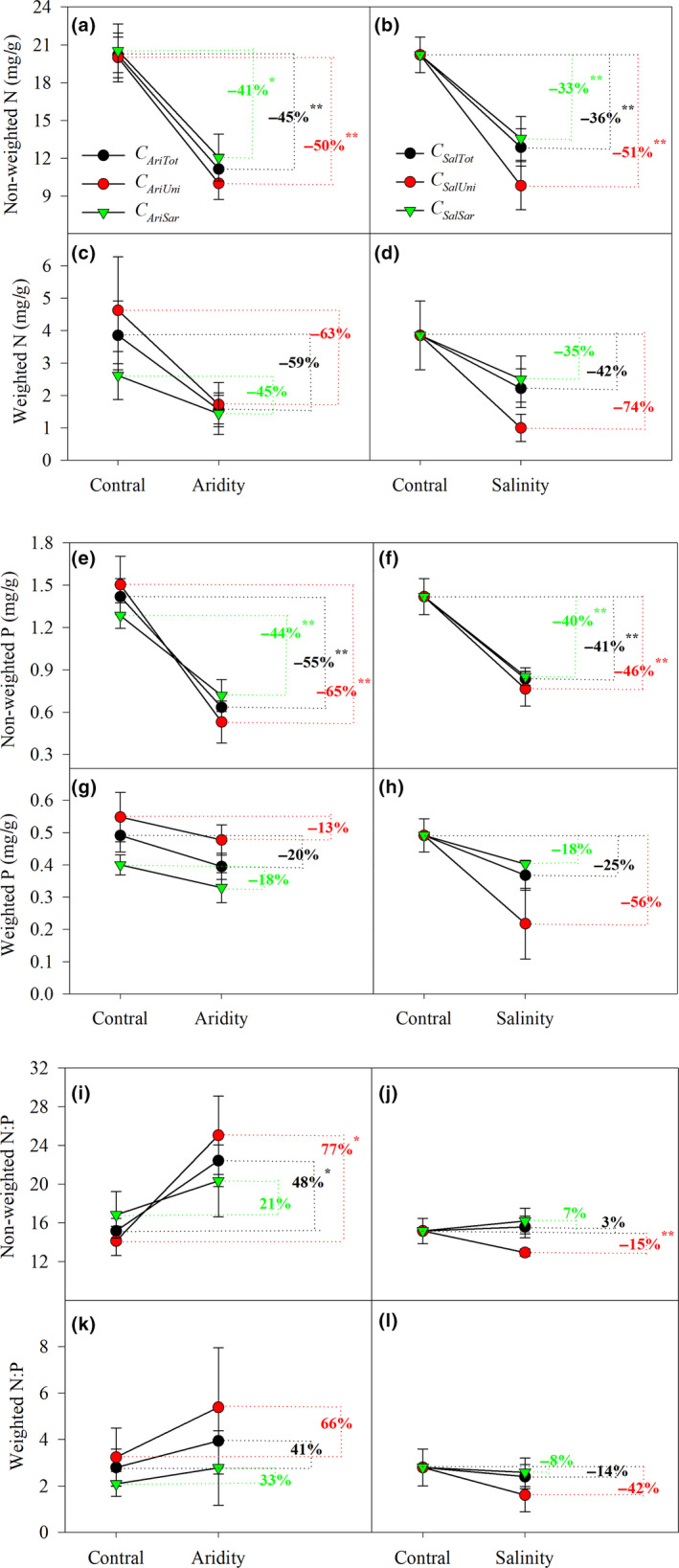
Changes in community [N], [P], and N:P in response to drought and salinity in local dryland vegetation. This figure shows the response of community stoichiometry estimated to be due to unique species and shared species variation for aridity and salinity filtering. *C_AriUni_*, *C_AriSha_*, and *C_AriTot_* represent the isolated effects due to the shifts of unique species, shared species, and total species, respectively, in driving the foliar nutritional responses to aridity filtering. This description is also consistent with *C_SalUni_*, *C_SalSha_*, and *C_SalTot_* under salinity filtering. *p < .05. **p < .01

**FIGURE 3:**
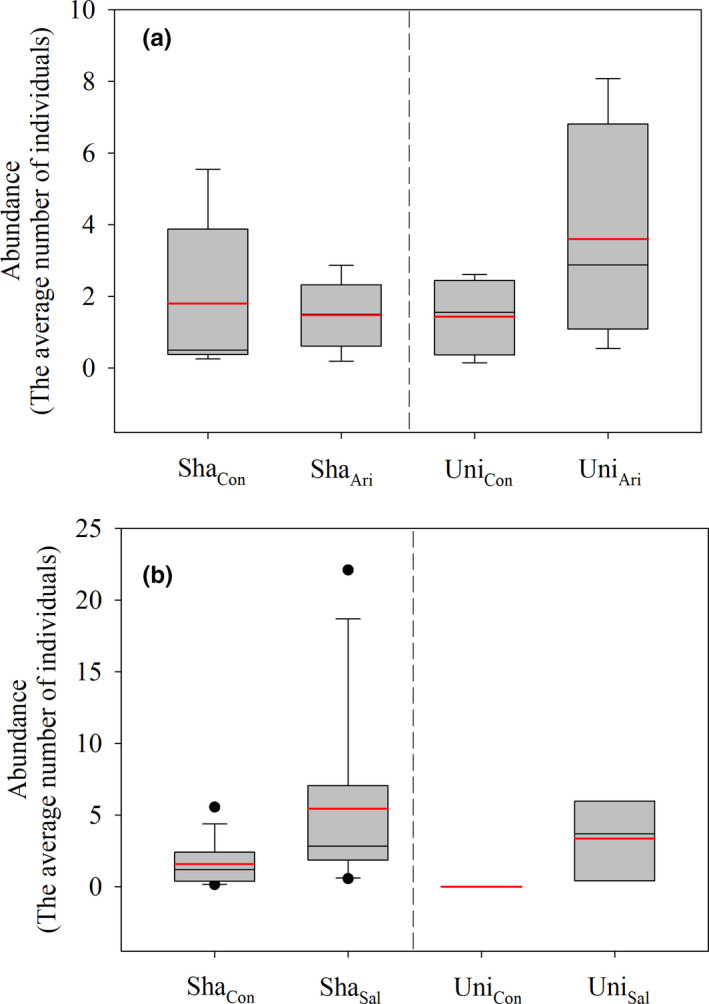
The abundance of plant individuals from unique and shared species in the control, aridity and salinity sites, respectively. Abundance, the average number of individuals per 100 square meters. The red solid line indicates average value. Sha_Con_, abundance of shared species in the control sites. Sha_Ari_, abundance of shared species in the aridity sites. Uni_Con_, abundance of unique species in the control sites. Uni_Ari_, abundance of unique species in the aridity sites. Sha_Sal_, abundance of shared species in the salinity sites. Uni_Sal_, abundance of unique species in the salinity sites

The analysis of changes in community composition showed no significant difference in species richness among the control, arid, and saline habitats (Figure S4). Similarly, there was no significant difference in the abundance of individual plants from unique and shared species in the control, arid, and saline sites (Figures 2, 3).

### 3.2 Changes in N, P, and N:P in unique and shared species in response to aridity and salinity filtering

CM of N in both arid and saline habitats was significantly lower than in the control habitat (45% and 36%, respectively; *p* < .01, Figure 2a,b). Unique and shared species were relative changes of 50% and 41% to the nonweighted N in the arid habitats, and 51% and 33% in the saline habitats, respectively. Similarly, under aridity and salinity filtering, the weighted N of unique species and shared species decreased by 63% and 45% (in the arid habitat) and by 74% and 35% (in the saline habitat), respectively, resulting in a decrease in the overall community N of 59% and 42%, respectively, although this was not significantly (Figure 2c,d).

Driven by aridity and salinity stress, the nonweighted P of all the species on the community scale was significantly lower than in the control habitat (*p* < .01, Figure 2e,f). Addressing the relative contribution of unique and shared species, the nonweighted P of unique and shared species was significantly reduced (by 65% and 44%, respectively) in the arid habitat, by 46% and 40%, respectively in the saline habitat compared with the control habitat (*p* < .01, Figure 2e,f). The weighted P of unique and shared species decreased by 13% and 18% in the arid habitat, and by 18% and 56% in the saline habitat compared with the control site (Figure 2g,h), the decreases are not significant (*p* > .05).

In contrast, inconsistent results were found regarding shifts in community‐level N:P ratios under environmental change (Figure 2i–l). Aridity caused significant increases in nonweighted (*p* < .05) and weighted N:P ratios in both unique (by 77% and 21%, respectively) and shared species (by 66% and 33%, respectively; Figure 2i,k). However, the effects of salinity stress on nonweighted and weighted N:P ratios of the shared species were less than on those of the unique species (22% and 50% lower, respectively, Figure 2j,l), but not significantly (*p* > .05).

## 4 DISCUSSION

In the analysis of community‐level N:P stoichiometry, we combined data from unique and shared species (i.e., within‐ and among‐species variability) from different communities under the influence of environmental change (Teurlincx et al., 2017). We found that communities with high interspecific variation, reflecting large stoichiometric differences among species, were more likely to include species with stoichiometry preadapted to drought and salinity stresses, facilitating rapid shifts in community stoichiometric composition through species turnover (Siefert & Ritchie, 2016). Therefore, separating the stoichiometric information of unique and shared species, our approach allowed us to address the extent of community‐level N:P stoichiometric plasticity and to evaluate the impact of its major underlying drivers (i.e., organism plasticity, and community assembly processes, such as species losses and gains and changes in the relative abundance of species; Teurlincx et al., 2017). This indicated an decrease in the stoichiometric traits of shared species with higher aridity and salinity stresses. In addition, plant species with high nutrient content were replaced by species with lower nutrient concentrations under aridity and salinity filtering. Nutrient concentrations of the plant communities were characterized by abrupt shifts in the losses or gains of the unique species (Tables 1 and 2), rather than by gradual changes resulting from stoichiometric variation in the shared species under either salinity or aridity stress (i.e., Figure 2; Costa et al., 2017). Therefore, our study shows that the decrease in N:P stoichiometry caused by species turnover driven by drought and salt stress can be divided into two parts: one is the decrease in individual stoichiometry of shared species, and the other is the decrease caused by species replacement.

Based on the CW and CWM calculations, our results confirm that community stoichiometric changes are higher in species turnover of unique species than in shared species (Violle et al., 2007, 2012; Zuo et al., 2016). Consequently, comparing the responses of the CW and CWM to the habitat changes helps us to understand the role of subdominant and dominant species (Lv et al., 2018). Our results indicated that the use of CM and CWM did not alter the results for the fluctuations in foliar N and P concentrations and N:P ratios between control, arid, and saline plots (Figure 2). Compared with the changes in CM, CWM showed that changes in the N concentration of both unique and shared species were reduced under aridity and salinity filtering (Figure 2). In addition, CWM increased the changes in P concentrations for unique species under salinity stress, and reduced them for unique and shared species compared with those changes under CM driven by aridity and salinity stresses. Surprisingly, CWM and CM had the opposite effect on N:P ratios of unique species under aridity and salinity stress. For example, drought caused an increase in the N:P ratios of unique species (Figure 2i,k), but a decrease in salinity caused the N:P ratios of unique species (Figure 2j,l). These results suggest that the CWM is a more ecologically sound calculation, not only considering the presence of each constituent species, but taking into account their relative contribution to the entire community in terms of biomass or dominance. For both CWM and CM, plant foliage N and P concentrations decreased with increasing aridity (or salinity) at the community level, primarily because of the larger effects of species turnover from changes in unique species compared with those from plasticity of shared species (Figure 3). Our results are in agreement with Luo et al. (2018), who reported provided that species turnover has a major role in changes in canopy nutrient concentrations in response to long‐term aridity.

Plant communities can respond to environmental changes by altering the plasticity and adaptability of their N:P stoichiometry at intraspecific level and by species turnover at the interspecific level (Ackerly, 2004; Amatangelo, Johnson, Rogers, & Waller, 2014; Jung et al., 2014). In desert ecosystems, the optimal survival strategy for plants is to reduce their growth and metabolic rate, as well as their transpiration and photosynthetic rates in response to extreme aridity or high salinity stresses (Jager, Richardson, Bellingham, Clearwater, & Laughlin, 2015). Patterns of plant nutrient allocation and stoichiometry reflect the use of N and P to sustain plant metabolism and growth (He et al., 2006; Yuan & Chen, 2015). Thus, plant communities adapt to harsh environments, such as desert ecosystems, by reducing their N:P stoichiometry. Consistent with our conclusions, species associated with low fertility soils had comparatively “slower” leaves with low leaf N and P concentrations compared with species grown in high fertility soils (Jager et al., 2015). However, our findings are inconsistent with the studies reporting higher plant nutrient concentrations with increasing aridity in grassland systems (Luo et al., 2016, 2018; Sandel et al., 2010). For grassland ecosystems, plant community assembly tends to select species with higher nutrient concentrations to increase their competitive advantage under persistent water stress (Li et al., 2017; Luo et al., 2018; Sardans & Penuelas, 2012). Plants can opportunistically maximize their photosynthesis by increasing their foliage nutrient concentrations, thus improving their water use efficiency and their ability to cope with aridity (Liu et al., 2010; Weih, Bonosi, Ghelardini, & Ronnberg‐Wastljung, 2011; Wright & Westoby, 2002).

## 5 CONCLUSIONS

In summary, the low nutrient stoichiometric species in our arid and saline plant community were likely to have been selected because they are more adaptable to extreme aridity and high salinity habitats. Furthermore, such environmental changes can cause the loss of the plant species with high nutrient concentrations and low plasticity, but the gain of species with either low nutrient concentrations (from unique species), high stoichiometric plasticity (from shared species), or both. In addition, the important roles of dominant species in the stoichiometric changes in the community were highlighted by the weighted community‐average values of relative abundance. Thus, our results revealed the plant community construction mechanism in extreme environments in arid areas by separating the relative contributions of unique and shared species to the stoichiometric variation of the community.

## CONFLICT OF INTEREST

None declared.

## AUTHOR CONTRIBUTION

**Yan‐Ming Gong:** Conceptualization (equal); Data curation (equal); Formal analysis (equal); Investigation (equal); Methodology (equal); Resources (equal); Software (equal); Supervision (equal); Validation (equal); Writing‐original draft (equal); Writing‐review & editing (equal). **Guang‐Hui Lv:** Conceptualization (lead); Funding acquisition (lead); Project administration (lead); Resources (equal); Supervision (equal); Visualization (equal). **Hong‐Bo Ling:** Data curation (equal); Resources (equal); Software (equal); Validation (equal). **Yue Chen:** Data curation (equal); Investigation (equal); Methodology (equal). **Jing Cao:** Data curation (equal); Formal analysis (equal); Investigation (equal); Resources (equal). **Zhen‐Jie Guo:** Data curation (equal); Investigation (equal); Methodology (equal).

## Supporting information

Figs S1‐S4

## Data Availability

Data from this study are available and can be accessed at the public data repository Dryad.
