# Supplementary material for: N:P stoichiometric changes via species turnover in arid versus saline desert environments
Source: Ecol Evol. 2020 May 30;10(13):6636–45. doi: 10.1002/ece3.6395 (PMC7381577; doi:10.1002/ece3.6395)
Supplement: Supplementary file 1 — Figs S1‐S4 [file ECE3-10-6636-s001.pdf]

N:P stoichiometric changes via species turnover in arid versus saline desert environments

Appendix:

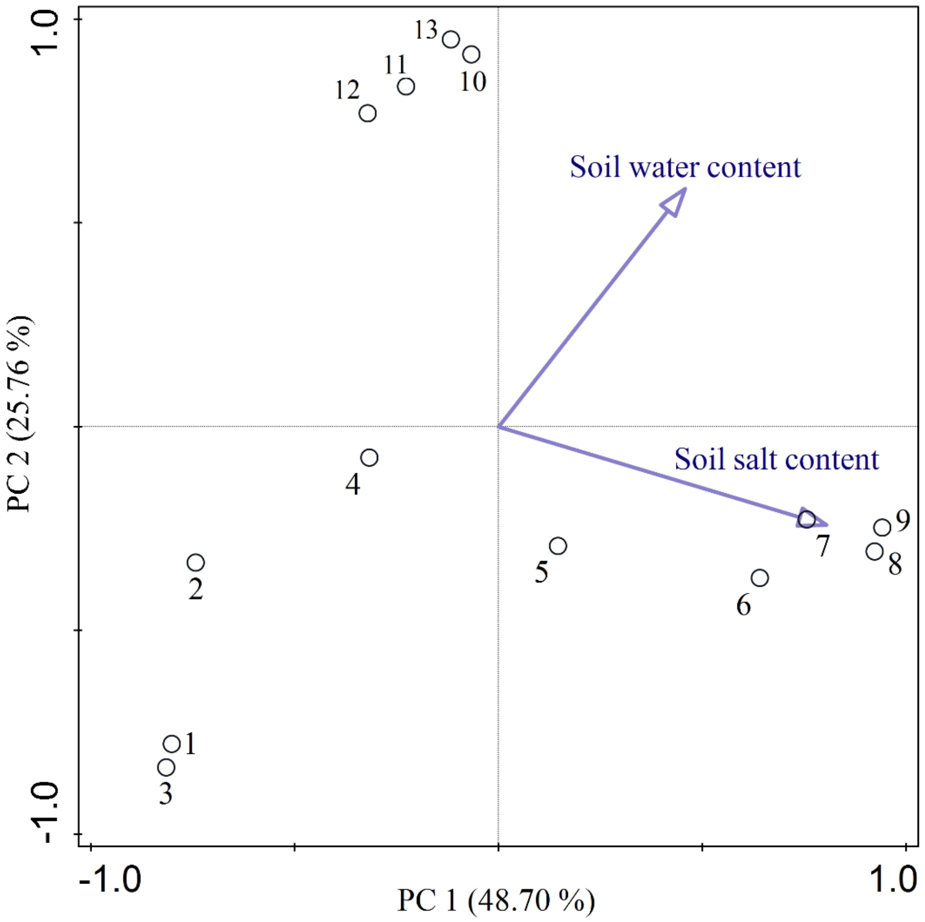

Figure S1. A principal component analysis (PCA) for the thirteen sites by the soil water and salt content. Sites 1, 2, 3 and 4, sites 5, 6, 7, 8, and 9, sites 10, 11, 12 and 13 were defined aridity sites, salinity sites and control sites, respectively.

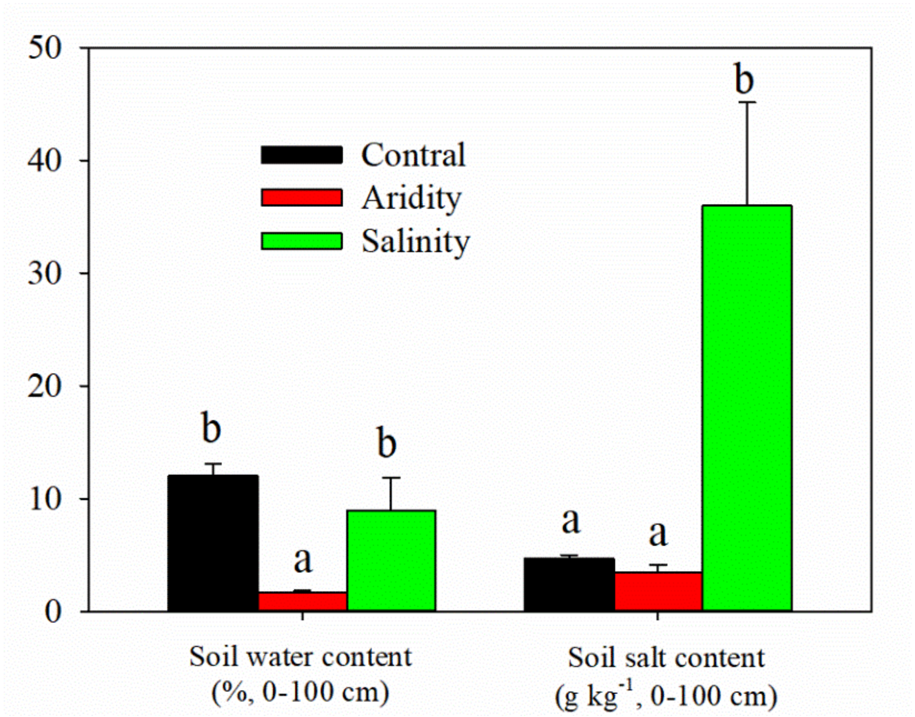

Figure S2. Values of soil water content and salt content measured at control, aridity and salinity sites along a natural gradient in the arid local vegetation. Lowercase letters indicate significant differences ($P < 0.05$) in control, aridity and salinity sites.

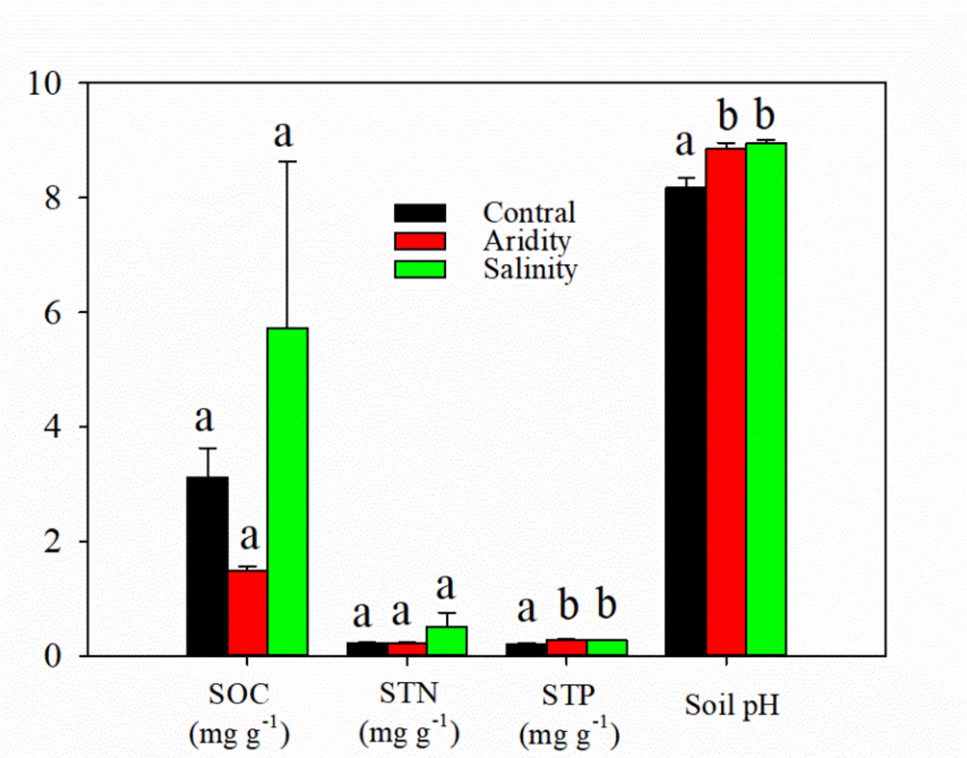

Figure S3. Values of soil organic carbon (SOC), soil total nitrogen (STN), soil total phosphorus (STP) and soil pH measured at control, aridity and salinity sites along a natural gradient in the arid local vegetation. Lowercase letters indicate significant differences ($P < 0.05$) in control, aridity and salinity sites.

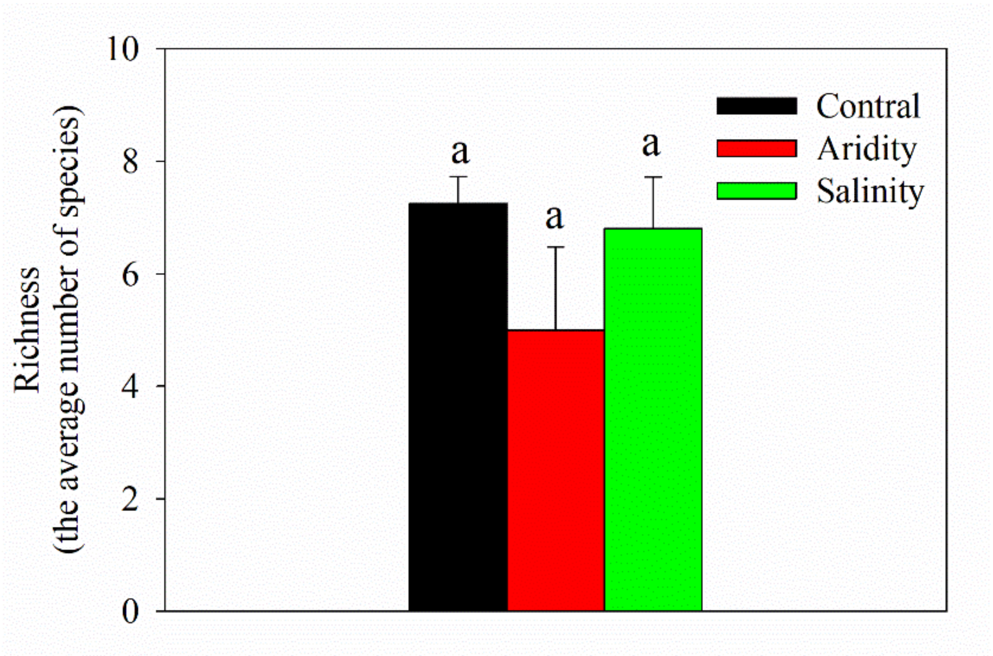

Figure S4. Values of richness (the average number of species per 100 square meters) measured at control, aridity and salinity sites along a natural gradient in the arid local vegetation. Lowercase letters indicate significant differences ($P < 0.05$) in control, aridity and salinity sites.
